# Supplementary material for: Adaptation of health systems to climate change-related infectious disease outbreaks in the ASEAN: Protocol for a scoping review of national and regional policies
Source: PLoS One. 2023 Jun 6;18(6):e0286869. doi: 10.1371/journal.pone.0286869 (PMC10243619; doi:10.1371/journal.pone.0286869)
Supplement: S1 Fig — (DOCX) [file pone.0286869.s001.docx]

**Supporting Information**

**S1 Fig. Dengue fever and malaria transmission in ASEAN countries, assumming high emission scenario.** (a) Mean vectorial capacity for dengue fever transmission, defined as the vector (Aedes aegypti) propensity to transmit dengue to human, in ASEAN countries, assuming high emission scenario. Note that the projected increase of mean vectorial capacity, while variable in number, is observed in all reported countries. Reports on Singapore, Laos, and Vietnam are not available. (b) Population at risk of malaria in ASEAN countries, assuming high emission scenario. The projected increase is observed in all reported countries, with the largest increase observed in Indonesia (almost two-fold). Reports on Brunei Darussalam, Singapore, Laos, and Vietnam are not available. For both situation, similar condition is observed in low emission scenario, with slightly lower increase. [5–11]
