# Supplementary material for: Adaptation of health systems to climate change-related infectious disease outbreaks in the ASEAN: Protocol for a scoping review of national and regional policies
Source: PLoS One. 2023 Jun 6;18(6):e0286869. doi: 10.1371/journal.pone.0286869 (PMC10243619; doi:10.1371/journal.pone.0286869)
Supplement: S1 Checklist — (DOCX) [file pone.0286869.s002.docx]

**Preferred Reporting Items for Systematic reviews and Meta-Analyses extension for Scoping Reviews (PRISMA-ScR) Checklist**

| **SECTION** | **ITEM** | **PRISMA-ScR CHECKLIST ITEM** | **REPORTED ON PAGE #** |
| --- | --- | --- | --- |
| **TITLE** | | | |
| Title | 1 | Adaptation of health systems to climate change-related infectious diseases outbreaks in the ASEAN: Protocol for a scoping review of national and regional policies | Title page |
| **ABSTRACT** | | | |
| Structured summary | 2 | Abstract provided | 2 |
| **INTRODUCTION** | | | |
| Rationale | 3 | Describe the rationale for the review in the context of what is already known. Explain why the review questions/objectives lend themselves to a scoping review approach. | 3 |
| Objectives | 4 | Aim: To map the evidence of climate-related health adaptation policies and programs in the ASEAN, both at the country level and within the ASEAN as a regional body, with particular focus on policies related to infectious diseases control.  Research question: How climate change adaptation are included into health policies and actions in the ASEAN health systems, with particular focus on infectious diseases control?  Sub-questions:   1. What are the characteristics of existing policies and actions for climate change adaptation in health systems among ASEAN member states and the ASEAN region, with particular focus on infectious diseases control? 2. To what extent do the current climate-related health policy documents from AMS adopt the WHO Operational Framework for Climate-Resilient Health Systems? 3. What are the indicators that have been used to assess for climate change adaptation in health systems among ASEAN member states and the ASEAN region, with particular focus on infectious diseases control?   Eligibility assessment of the research questions   - P-Population: Humans. - C-Concept: Climate change adaptation policies and actions in health systems, focusing on infectious diseases & outbreaks. - C-Context: ASEAN member states (AMS) and the ASEAN | 3, 4 |
| **METHODS** | | | |
| Protocol and registration | 5 | This is a protocol submission | N/A |
| Eligibility criteria | 6 | Inclusion criteria:   1. Policy documents and studies on climate change adaptation policies and actions, with particular focus on infectious diseases, in ASEAN health systems. 2. Published within 20 years (between January 2003 to January 2023). If there are two or more policies published by a country or in ASEAN, we will only include policies that are still in effect.   Exclusion criteria: Conference proceedings, books, blogs, news articles, and articles that are not available in full-text form | 5 |
| Information sources* | 7 | ASEAN Secretariat website, government websites, Google, and six research databases (PubMed, ScienceDirect, Web of Science, Embase, WHO Institutional Repository Information Sharing (IRIS), and Google Scholar) | 5 |
| Search | 8 | Search strategy provided | S1 Appendix |
| Selection of sources of evidence† | 9 | Two reviewers will independently assess the eligibility and inclusion of the studies by screening the titles and abstracts, followed by full-text assessments. | 5 |
| Data charting process‡ | 10 | A form developed by the authors based on the WHO ten key components for building climate resilience | 5-6 |
| Data items | 11 | 1. Country 2. Name of documents 3. Type of documents 4. Issuing body 5. Type of addressed infectious diseases-related issues 6. Concordance to WHO’s ten key components for building climate resilience    1. Leadership and governance    2. Health workforce    3. Vulnerability, capacity & adaptation assessment    4. Integrated risk monitoring & early warning    5. Health & climate research    6. Climate resilient and sustainable technologies & infrastructure    7. Management of environmental determinants of health    8. Climate-informed health programs    9. Emergency preparedness and management    10. Climate and health financing | 6-7 |
| Critical appraisal of individual sources of evidence§ | 12 | N/A | N/A |
| Synthesis of results | 13 | To summarize our search results, we will use the PRISMA flow chart. To analyze the included policies, we will use the framework obtained from the WHO’s ten key components for building climate-resilient health systems | 5-7 |
| **RESULTS** | | | |
| Selection of sources of evidence | 14 |  | Click here to enter text. |
| Characteristics of sources of evidence | 15 |  | Click here to enter text. |
| Critical appraisal within sources of evidence | 16 | Click here to enter text. |  |
| Results of individual sources of evidence | 17 |  | Click here to enter text. |
| Synthesis of results | 18 | Click here to enter text. |  |
| **DISCUSSION** | | | |
| Summary of evidence | 19 | This data will be the basis for a summary narrative discussing the current national and regional policies on the topic, the infectious diseases that are the focus of these policies, and the remaining gaps that need to be addressed. | 6 |
| Limitations | 20 | The scoping review used English keywords used for the literature search, reducing the possibility of including articles in other languages. Furthermore, the year criteria (20 years) may also limit the included policies, although this is intended to give focus to current policies. | 6 |
| Conclusions | 21 | Click here to enter text. |  |
| **FUNDING** | | | |
| Funding | 22 | The author(s) received no specific funding for this work. | Click here to enter text. |

JBI = Joanna Briggs Institute; PRISMA-ScR = Preferred Reporting Items for Systematic reviews and Meta-Analyses extension for Scoping Reviews.

* Where *sources of evidence* (see second footnote) are compiled from, such as bibliographic databases, social media platforms, and Web sites.

† A more inclusive/heterogeneous term used to account for the different types of evidence or data sources (e.g., quantitative and/or qualitative research, expert opinion, and policy documents) that may be eligible in a scoping review as opposed to only studies. This is not to be confused with *information sources* (see first footnote).

‡ The frameworks by Arksey and O’Malley (6) and Levac and colleagues (7) and the JBI guidance (4, 5) refer to the process of data extraction in a scoping review as data charting*.*

§ The process of systematically examining research evidence to assess its validity, results, and relevance before using it to inform a decision. This term is used for items 12 and 19 instead of "risk of bias" (which is more applicable to systematic reviews of interventions) to include and acknowledge the various sources of evidence that may be used in a scoping review (e.g., quantitative and/or qualitative research, expert opinion, and policy document).

*From:* Tricco AC, Lillie E, Zarin W, O'Brien KK, Colquhoun H, Levac D, et al. PRISMA Extension for Scoping Reviews (PRISMAScR): Checklist and Explanation. Ann Intern Med. 2018;169:467–473. [doi: 10.7326/M18-0850](http://annals.org/aim/fullarticle/2700389/prisma-extension-scoping-reviews-prisma-scr-checklist-explanation).
