# Supplementary material for: Adaptation of health systems to climate change-related infectious disease outbreaks in the ASEAN: Protocol for a scoping review of national and regional policies
Source: PLoS One. 2023 Jun 6;18(6):e0286869. doi: 10.1371/journal.pone.0286869 (PMC10243619; doi:10.1371/journal.pone.0286869)
Supplement: S1 Appendix — (DOCX) [file pone.0286869.s003.docx]

**S1 Appendix. Database search strategy.**

We will conduct a systematic search on the ASEAN Secretariat website, government websites, Google, as well as six research databases (PubMed, ScienceDirect, Web of Science, Embase, World Health Organization (WHO) Institutional Repository Information Sharing (IRIS), Google Scholar) to identify relevant policy documents and grey literature. In the ASEAN secretariat website, all posts from 2003-2023 will be checked to ensure no relevant articles are missed.

| **Database** | **Keywords** |
| --- | --- |
| ASEAN Secretariat website, government websites, Google | Various combinations of “Climate change”, “healthcare system”, “infectious diseases”, “ASEAN” |
| Pubmed | ("climate change"[MeSH Terms] OR ("climate"[All Fields] AND "change"[All Fields]) OR "climate change"[All Fields]) AND ("delivery of health care"[MeSH Terms] OR ("delivery"[All Fields] AND "health"[All Fields] AND "care"[All Fields]) OR "delivery of health care"[All Fields] OR ("healthcare"[All Fields] AND "system"[All Fields]) OR "healthcare system"[All Fields]) AND ("asia, southeastern"[MeSH Terms] OR ("asia"[All Fields] AND "southeastern"[All Fields]) OR "southeastern asia"[All Fields] OR ("southeast"[All Fields] AND "asia"[All Fields]) OR "southeast asia"[All Fields]) |
|  | ("climate change"[MeSH Terms] OR ("climate"[All Fields] AND "change"[All Fields]) OR "climate change"[All Fields]) AND ("delivery of health care"[MeSH Terms] OR ("delivery"[All Fields] AND "health"[All Fields] AND "care"[All Fields]) OR "delivery of health care"[All Fields] OR ("healthcare"[All Fields] AND "system"[All Fields]) OR "healthcare system"[All Fields]) AND ("asia, southeastern"[MeSH Terms] OR ("asia"[All Fields] AND "southeastern"[All Fields]) OR "southeastern asia"[All Fields] OR ("southeast"[All Fields] AND "asia"[All Fields]) OR "southeast asia"[All Fields]) AND infectious diseases |
| ScienceDirect | Climate change AND infectious diseases AND healthcare system AND (ASEAN OR "southeast Asia") (article type: research article) |
| Web of Science | Climate change AND healthcare system AND (ASEAN OR southeast Asia OR Malaysia OR Indonesia OR Brunei OR Cambodia OR Myanmar OR Singapore OR Laos OR Philippines OR Thailand OR Vietnam) AND infectious diseases |
| Embase | Climate change AND healthcare system AND (ASEAN OR southeast Asia OR Malaysia OR Indonesia OR Brunei OR Cambodia OR Myanmar OR Singapore OR Laos OR Philippines OR Thailand OR Vietnam) AND infectious diseases |
| World Health Organization (WHO) Institutional Repository Information Sharing (IRIS) | Climate change AND healthcare system AND (ASEAN OR southeast Asia OR Malaysia OR Indonesia OR Brunei OR Cambodia OR Myanmar OR Singapore OR Laos OR Philippines OR Thailand OR Vietnam) AND infectious diseases (Filter applied: Climate change [Title]; date published from 2000-2023) |
| Google Scholar | Climate change AND healthcare system AND (ASEAN OR southeast Asia OR Malaysia OR Indonesia OR Brunei OR Cambodia OR Myanmar OR Singapore OR Laos OR Philippines OR Thailand OR Vietnam) AND infectious diseases |
